# Increased risk of Graves´ophthalmopathy in patients with increasing TRAb after radioiodine treatment and the impact of CTLA4 on TRAb titres

**DOI:** 10.1007/s12020-021-02952-2

**Published:** 2021-12-02

**Authors:** Bushra Shahida, Kleoniki Tsoumani, Tereza Planck, Vijayachitra Modhukur, Pernilla Asp, Anna Sundlöv, Jan Tennvall, Peter Åsman, Ola Lindgren, Mikael Lantz

**Affiliations:** 1 Department of Clinical Sciences Malmö, Diabetes and Endocrinology, Lund University, Malmö, Sweden; 2 Department of Endocrinology, Department of Clinical Sciences, Skåne University Hospital, Lund University, Malmö and Lund, Sweden; 3 Department of Oncology, Skåne University Hospital, Lund, Sweden; 4 Department of Oncology, Department of Clinical Sciences, Skåne University Hospital, Lund University, Lund, Sweden; 5 Department of Clinical Sciences Malmö, Ophthalmology, Skåne University Hospital, Department of Ophthalmology, Lund University, Malmö, Sweden

**Keywords:** Graves´ disease, TRAb, Anti-TPO, Anti-TG, Radioiodine, CTLA-4

## Abstract

**Introduction:**

Treatment of Graves´ disease (GD) with radioiodine increases the risk of developing Graves´ ophthalmopathy (GO), and the link between thyroid and orbital tissue may be the presence of TSH-receptors. Radioiodine increases the titers of TRAb and the aim was to investigate the relationship between GO and TRAb titers after treatment with radioiodine and to define the impact of risk genes.

**Methods:**

GD patients without ophthalmopathy or previous treatment with radioiodine were prospectively included at treatment with radioiodine for hyperthyroidism. A follow-up was performed 1 year later for the registration of GO development. The study was performed at a University Hospital Clinic; a referral center of all patients treated with radioiodine in the south of Sweden. The main outcome measures were the development of TRAb, anti-TPO, and anti-TG after 3 months and GO after 12 months and relationship to the genetic background (HLA, CTLA-4, and CYR61).

**Results:**

Three months of radioiodine TRAb titers increased in two thirds of patients (*p* < 0.0005) but not in the other third. Anti-TPO titers were associated with TRAb (*R* = 0.362, *p* < 0.0001) but not anti-TG. At follow-up 1 year later (*n* = 204) 32 patients developed GO with a proportion of 70% in the group increasing in TRAb titers and 30% in the group with unchanged or lower TRAb titers (*p*-value < 0.0005). Patients with GO had higher titers of TRAb than patients without GO. CTLA-4 (rs231775 SNP) was significantly (*p* < 0.005) associated with TRAb titers above the median three months after radioiodine.

**Conclusions:**

The increase in TRAb titers after treatment with radioiodine is associated with GO and a genetic variation in CTLA-4 is associated with higher titers of TRAb.

## Introduction

In Graves´ disease (GD), immunocompetent cells infiltrate thyroid tissue with the release of TSH-receptor stimulating antibodies (TRAb), resulting in hyperthyroidism. The triggering of the autoimmune response depends on the interplay of genetic [1, 2] and environmental factors [3]. A strong risk factor is tobacco smoking which results in higher TRAb at diagnosis of GD and during treatment with thiamazole than non-smokers [3, 4].

Smoking has been shown to increase TRAb titers and the risk of development of GO both at and after diagnosis of GD [5, 6]. Treatment of GD with radioiodine is a risk factor for the development of GO and might be mediated by activating TRAb [7]. Laurberg et al. has shown that treatment with radioiodine increases TRAb with a maximum after 3 months which is in contrast to treatment with anti-thyroid drugs (ATD) or thyroidectomy, where TRAb slowly declines without a prior increase [8]. It has been suggested that the increase in TRAb after radioiodine treatment is mediated by a transient release of thyroid antigens [9, 10]. However this assumption has been difficult to confirm. In some patients the increase in TRAb persists for several years, indicating the existence of other mechanisms involving the activation of specific immunocompetent cells or a prolonged effect of radioiodine on immunocompetent cells. Irradiated Hashimoto lymphocytes have been studied in vitro, and it was proposed that irradiated lymphocytes of the thyroid are important insynthesising of autoantibodies in response to 131-Iodine [11].

We have previously identified immediate early genes (IEGs) overexpressed in orbital tissue from patients with severe GO [12]. These genes have later been investigated for the presence of gene polymorphisms and we found an association of CYR61 with GD and GO, and an increased risk for GO in smokers (OR 4.75) [13]. Later, in a microarray analysis of human orbital tissue, we showed that the IEGs exhibited higher expression in smokers than non-smokers [14]. Cytotoxic T-lymphocyte antigen (CTLA-4) and human leukocyte antigen (HLA) are other genes with known association to GD [2]. There are no known genes associated only with GO.

In a previous study on the treatment of GD patients with radioiodine we have defined one group with increases in TRAb, thyroid peroxidase antibodies (anti-TPO), thyroglobulin antibodies (anti-TG), and another group with no increase of these thyroid antibodies after treatment with radioiodine [15]. We have now increased the number of patients and followed these patients for at least 1 year.

The present study aimed to investigate the relationship between TRAb and GO development and define the impact of risk genes.

## Material and methods

### Subjects and treatment

Treatments with 131-iodine in Skåne County are centralized to the department of Oncology in Lund where all patients with hyperthyroidism are referred if the patient´s ordinary clinician decides that radioiodine is the best treatment choce.

This prospective observational study included GD patients, without GO, admitted to the Department of Oncology for treatment with radioiodine from August 2016 until August 2018 (*n* = 204) and followed up 1 year later for the development of GO

TRAb, anti-TPO, and anti-TG were measured before and 3 months after 131-iodine treatment and the fold changes were calculated.

Patients without GO at radioiodine treatment but with risk factors for the development of GO were prescribed (by the treating clinician), prophylaxis with prednisolone. These patients (*n* = 45) received prednisolone 30 mg per day, independently of body weight, for 1 month and after that the dose was slowly decreased during the next 2 months and stopped after 3 months.

The patients were classified as having GD based on clinical signs, increased thyroid hormones and suppressed TSH, the presence of TRAb, and/or a diffuse uptake on thyroid technetium scintigraphy; all patients performed this investigation. One year, later all patients were screened for the GO development (or not) by an endocrinologist or an ophthalmologist at their home clinic in Skåne. All patients received a questionnaire and DNA swab for self-collection of DNA from the buccal mucosa which 130 out of 204 patients accepted and sent in an envelope to our research laboratory. DNA was amplified and analysed for the presence of polymorphisms in known risk genes for GD and GO; cytotoxic T-lymphocyte antigen 4 (CTLA4), human leukocyte antigen-DRB (HLA-DRB) and, cysteine-rich 61 (CYR61).

In total 204 patients not previously treated with radioiodine were included. The following parameters were registered; age, sex, born in Sweden, tobacco smoking, duration of GD and GO, and treatment with corticosteroids (Table 1).

**Table 1:** Changes in TRAb after treatment with radioiodine and relation to clinical parameteters

Fold change TRAb	<1.1	≥1.1	*P* value
Patients (%)	57 (31)	125 (69)	
Females (%)	45 (79)	97 (78)	0.84
Age males and females, years	56 ± 17	54 ± 16	0.67
Age females, years	54 ± 17	52 ± 16	0.49
Smokers (%)	8 (14)	31 (25)	0.10
Born outside Sweden (%)	12 (21)	35 (28)	0.32
Treatment with Corticosteroids (%)	41 (73)	79 (63)	0.23
Treatment with >120 Gy	10 (18)	21 (16)	0.99
Duration of GD, months	12 (5–25)	8 (3–33)	0.48
Primary treatment with ATD (%)	37 (65)	70 (56)	0.33
Duration of GO, months	6 ± 6	5 ± 3	0.52
Treatment with Corticosteroids in GO patients	2 (22)	4 (17)	0.99
GO smokers	0	2	0.99

The primary endpoint was development of GO and the number of patients planned to be included was approximately 200 patients to obtain approximately 40 ophthalmopathy patients, which is based on the knowledge of a previous Swedish multicenter study [7] where 20% developed ophthalmopathy during the first year. Based on our previous study on radioiodine where two thirds increased in TRAb and one third were unchanged we assumed that GO was distributed similarly, which should give a significant difference of ophthalmopathy between groups. The secondary endpoint was the presence of polymorphisms in known risk genes in patients with high TRAb.

As a clinical routine 120 Gray was used, but in some patients up to 300 Gray was administered if the aim was to decrease the risk of relapse. Methimazole or propylthiouracil with or without l-thyroxine was used in some patients before radioiodine and was stopped 2 weeks (1 week before the start of iodine uptake measurement) before administering of 131-iodine. The ALARA (as low as reasonably achievable) principle was used when defining the activity needed to achieve the described doses and the activity (MBq) was estimated by use of the following formula: Dose (D) × Mass (m)/0.043 × uptake day zero (U0) × effective half-life (Teff). U0 and Teff were calculated from the iodine uptake at 24 h and 7 days. The thyroid mass was calculated from 99mTc-pertechneate scintigraphy.

The proportion of absorbed doses used were distributed as follows: 120 Gy (78%), >120–300 Gy (22%).

### Gene polymorphisms analysis and DNA extraction

In total five single nucleotide polymorphisms (SNPs) were genotyped in 127 patients (Table 2); Two SNPs, rs1378228 and rs12656618, in CYR61 and two in CTLA4, rs3087243, and rs231775, were chosen for analysis based on previous studies on GD and GO (13) Also, a tag SNP, rs6457617, HLA Class II, DR *Beta 1* (HLA-DR-DQQ was analysed as previously described [16].

**Table 2:** Characteristics of genotyped patients

	TRAb (IU/L) < median (15 IU/L)	TRAb (IU/L) ≥ median (15 IU/L)
*N*	57	60
Age	55 ± 18	54 ± 15
*Sex*		
Male	13 (23)	15 (25)
Female	44 (77)	45 (75)
*Ethnicity*		
Born in Sweden	44 (77)	47 (78)
Born in Europe	7 (12)	7 (12)
Born outside Europe	6 (11)	6 (10)
Smokers	12 (21)	10 (17)
Non-smoker	41 (72)	39 (65)
Missing	4 (7)	11 (18)
*TRAb IU/L (median 15 IU/L)	6 (3–9)	31 (24–39)

Missing information on ten patients

Values expressed are means (±SD) and presented as *n* (%) unless otherwise stated

DNA was collected for genotyping using buccal swabs, then extracted using QiAamp UCP DNA Micro Kit (Qiagen, Sweden) and amplified using Repli-g Screening Kit (Qiagen, Sweden). SNPs were genotyped by TaqMan Allelic Discrimination Assay using the Quantstudio 7 Flex system (Applied Biosystems by Life Technologies, Sweden).

The minor allele frequency (MAF) for all SNPs was > 0.05. One SNP (rs12756618 in CYR61) failed the Hardy–Weinberg equilibrium and was excluded from the analysis.

The standard statistical analysis approach was used to find the association of TRAb < median/TRAb > median and GO/no GO association. A linear regression model was used with smoking and gender as covariates. The data are presented as odds ratios (ORs) with 95% confidence intervals (CIs). The *p*-values are based on additive models for the genetic variants. All genetic analyses were performed using PLINK version 1.0 (http://pngu.mgh.harvard.edu/~purcell/plink/index.shtml).

### Assays for antibodies

TRAb was measured with a competitive Electro Chem Luminiscens Immunoassay (ECLI) according to the manufactures instructions (Roche). The limit of detection was 0.3 IU/L, CV 5% at 16 IU/L. The cut-off for a positive value of TRAb was >1 kIU/L.

Anti-TPO titer was measured with a competitive sandwich ELISA (Roche) according to the manufactures instructions (detection limit 5 kIU/L, CV 11% at 34 kIU/L. The cut-off for a positive value of anti-TPO was >34 kIU/L.

Anti-TG was measured with a competitive sandwich ELISA (Roche) according to the manufactures instructions (detection limit 10 kIU/L, CV 10% at 73 kIU/L). The cut-off for a positive value of anti-TG was >115 kIU/L.

Samples were analysed in routine clinical laboratory at the Department of Clinical Chemistry in Malmö and Lund.

### Statistics

The fold changes of thyroid antibodies were calculated. A change of 1.1 or more was judged as an increase and if lower than 1.1 the change was judged as unchanged or decreased. The *t*-test (continuous variables), chi-square test (categorical variables), and bi-nomial test were used to assess the statistical significance of differences between the groups. Linear regression analysis was used to study the correlations between the parameters fold change of TRAb, anti, TPO, and anti-TG. All statistical analyses were carried out using the SPSS 22.0 statistical software (SPSS, Chicago, IL, USA) or Graph Pad prism 8.0 The significance levels were *p < 0.05; **p < 0.01; ***p < 0.001, ****p < 0.0001.

## Results

At the start of treatment with radioiodine 204 patients were registered and thyroid antibodies; TRAb, anti-TPO, anti TG were analysed before treatment with radioiodine, and 3 months after radioiodine the antibody analysis was repeated for detection of patients that showed an increase of 1.1 or more. The cut-off was set based on the knowledge of the variation coefficient for the antibody assays.

**Fig. 1:**
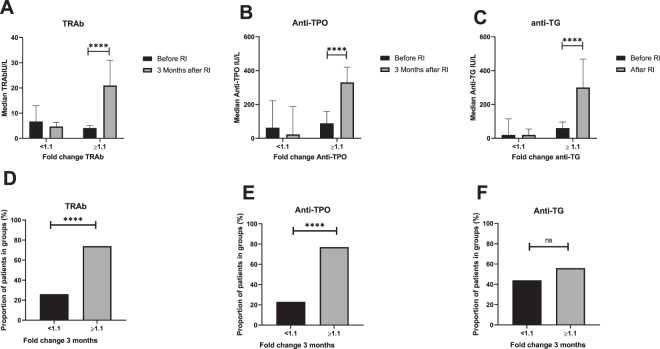
Fold change and proportion of TRAb (**A**, **D**), anti-TPO (**B**, **E**), and anti-TG (**C**, **F**) 3 months after treatment of Graves´ disease with radioiodine. The median values of TRAb, anti-TPO and anti TG before and after radioiodine in the group with fold change < 1.1 and in the group with fold change >/= 1.1 were all significant with *p*-values < 0.0001 (*t*-test). Differences in proportion was calculated with a binomial test and differences in median values with a *t*-test

**Fig. 2:**
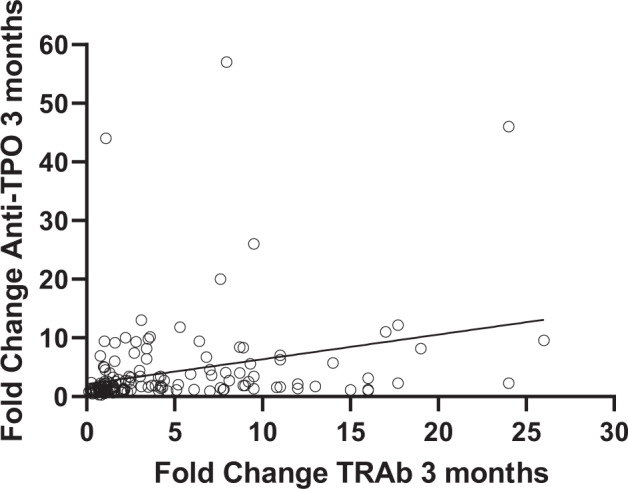
Correlation of fold change in TRAb and anti-TPO 3 months after treatment with RI (*R* = 0.362, *p* < 0.0001)

We found two groups according to antibody response 3 months after radioiodine; one group increased in titers of TRAb anti-TPO and anti-TG (70% of patients). Another group showed a decrease in titers of antibodies or were unchanged (30% of patients) (Fig. 1). There was not only a significant increase or decrease in fold change of antibody titers 3 months after radioiodine, but also a significant increase or decrease in median values of all three antibodies (Fig. 1). Three months after treatment, a correlation with radioiodine was found for titers of TRAb and anti-TPO (Fig. 2) but not for anti-TG (data not shown).

**Fig. 3:**
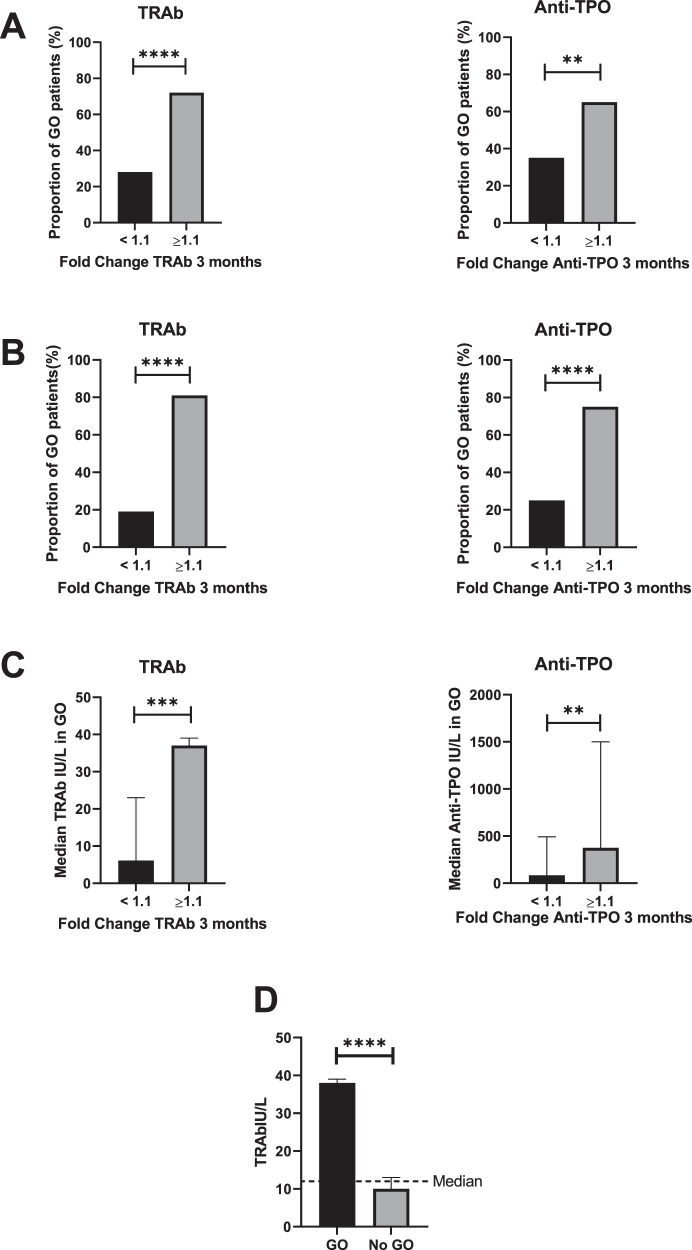
Development of Graves´ ophthalmopathy 1 year after RI in the whole group. **A** Increased TRAb (*n* = 32) or anti-TPO (*n* = 23) compared with no increase in TRAb or anti-TPO, 3 months after RI. Proportion of all GO patients in TRAb (*n* = 32) or anti-TPO (*n* = 23) including prednisolone treated patients in the group with no increase in antibodies. **B** Proportion of patients where prednisolone treated patients were excluded in the group with no increase in TRAb (*n* = 26) or anti-TPO (*n* = 20). **C** GO patients with median values of TRAb (*n* = 26) and anti-TPO (*n* = 20) in patients 3 months after radioiodine treatment. **D** Median values of TRAb in patients with and without GO with a dotted line that shows the median value of TRAb in all patients. Differences in proportion was calculated with a binomial test and differences in median values the *t*-test was used. Significance is shown in figures

All 204 patients were followed for at least 1 year after treatment with radioiodine to identify the development of GO and correlate GO to the antibody response. We found an overrepresentation of GO in the group with increased titers of TRAb and anti-TPO with a proportion of GO patients at follow-up after 1 year of 70% versus 30% in the group with no increase in antibody titers three months after radioiodine (Fig. 3A). If we excluded those patients with GO and low or unchanged titers of TRAb or anti-TPO (Fig. 3B) who had received treatment with prednisolone the effect of antibodies was more pronounced with a proportion of GO of 80% versus 20% for TRAb and similar proportions for anti-TPO (Fig. 3B). When analysing GO patients for TRAb and anti-TPO the median values were significantly different, with higher values in the group of GO patients with increased titers of TRAb compared with unchanged or lower values (Fig. 3C). If dividing the material in patients with or without GO the median TRAb titers were significantly higher in patients with GO and the majority of patients with GO have antibody titers above the median of the entire material (Fig. 3D).

We also tried to define an association of increase or decrease in antibody titers and clinical parameters previously associated with the incidence of GD, e.g., sex, ethnic background, smoking, and age, but we found no correlation with these parameters (Table 1). We also studied factors that might affect treatment with radioiodine (treatment dose, duration of disease, primary treatment) and found no factor that affected the antibody response (Table 1). Lastly we also studied factors that might increase the presence and risk of GO (smoking, duration of GO, treatment with steroids), these were not different between the antibody response groups (Table 1). The size of the thyroid was determined by technetium scintigraphy but did not affect the two defined groups (data not shown).

### Genotyping of Graves´ patients treated with radioiodine

We analysed SNPs in HLA, CTLA-4, and CYR61 in patients (*n* = 127) treated with radioiodine (Table 2) to define the genetic background in the development of GO and antibody response of TRAb, anti-TPO, and anti-TG. The response was divided into patients with increased or decreased antibody titers 3 months after radioiodine.

There was no significant difference in the presence of described SNPs between these groups nor did we detect a significant difference between patients with or without GO, although the allele frequency was lower in patients with GO (OR 0.56, *p* = 0.07) (Table 3). However, when analysing the material by dividing TRAb titers into groups above and below the median (15 IU/L) after treatment with radioiodine, the allele frequency of the rs231775 SNP in CTLA4 was significantly changed between groups (OR 0.48, *p* = 0.005) and associated with TRAb titers above the median which was confirmed by linear regression analysis of TRAb (data not shown), which was in contrast to anti-TPO and anti-TG which showed no significant association (Table 3).

**Table 3:** SNPs in GD patients treated with radioiodine.

Gene	SNP	Allele frequency TRAb (IU/L) < median (15 IU/L)	Allele Frequency TRAb (IU/L) ≥ median (15IU/L)	Associated Allele	OR for associated allele (95% CI)	*p-*value
*A. Association with TRAb below and above median 15 IU/L*						
CYR61	rs1378228	0.35	0.39	T	1.19 (0.72–1.99)	0.500
CTLA4	rs3087243	0.37	0.42	A	1.28 (0.77–2.12)	0.334
	rs231775	0.46	0.29	G	0.48 (0.28–0.80)	0.005
HLA-DRB1	rs6457617	0.42	0.42	C	1.30 (0.794–2.14)	0.295
*B. Association with GO*						
Gene	SNP	Allele frequency in patients without GO	Allele frequency in patients with GO	Associated allele	OR for associated allele (95% CI)	*p-*value
CYR61	rs1378228	0.36	0.4	T	1.181	0.58
CTLA4	rs3087243	0.37	0.48	A	1.62	0.10
	rs231775	0.41	0.28	G	0.56	0.07
HLA-DRB1	rs6457617	0.53	0.53	C	1.56	0.13

## Discussion

Strong risk factors for the development of GO are treatment with radioiodine and smoking, but mechanisms for these effects are, in most cases, not defined. The link between changes in thyroid tissue after radioiodine and orbital tissues is unknown.

We have shown that 3 months after treatment with radioiodine, two groups of patients were identified according to antibody response, one group that did not increase in titers of TRAb, anti-TPO, and anti-TG (approximately 30% of the patients) and one group that increased in antibody titers (approximately 70% of the patients). This has previously been demonstrated in a smaller group of GD patients [15].

The second observation was that the risk of developing GO we elevated in patients which increased in TRAb titers 3 months after radioiodine.

The third observation was that patients with a polymorphism in CTLA4 developed higher titers of TRAb than in patients without this polymorphism.

Laurberg et al. have previously shown that radioiodine treatment increases TRAb titers already 1 month after treatment and with a maximal response after 3 months [8]. We have identified two subgroups, one subgroup does not increase in antibody titers and the other group increased in thyroid antibody titers after treatment with radioiodine. The latter group might be important to identify because prednisolone is most often given to all risk patients at the start of radioiodine to prevent GO. Here we show that GO preferentially develops in the group that increases in thyroid antibody titers after 3 months indicating that this group should receive pre-treatment with prednisolone. In our study, we have treated risk patients with prednisolone at the start of radioiodine with a decrease of prednisolone 1 month later and with termination after 3 months when antibody titers are at maximal levels, and the prednisolone dose is low. Further studies are needed to define the kinetics of the increase in TRAb titers resulting in new routines for pre-treatment with prednisolone.

We and others have previously shown that treatment with radioiodine increases the risk of developing GO, but the mechanism has not been defined [7, 17, 18]. Here we show that the link between, the thyroid and the orbita tissue might be a release of TRAb and anti-TPO presumably from infiltrating immunocompetent cells of the thyroid, which later affect the orbital tissue in risk patients. One possible mechanism is that radioiodine decreases all immunocompetent cells, but the antibody producing cells have a higher proliferation rate than immunosuppressive cells, resulting in an imbalance between the described cells with an uncontrolled release of thyroid antibodies. In vitro it has been shown that a mixture of immunocompetent cells in Hashimoto produces thyroid specific antibodies after irradiation, and this effect was dose dependent [11]. We have previously studied the effect of the dose of radioiodine, but we could not confirm this in vivo [15].

Another hypothesis is that a transient increase in thyroid antigen like thyroglobulin will be released when irradiation destroys cells [9, 10]. However, we found no association between TRAb and thyroglobulin titers compared with TRAb and anti-TPO titers after treatment with radioiodine. Also in some cases thyroid antibodies might persist for several years after radioiodine indicating that other mechanisms exist.

Active smoking is another strong risk factor for autoimmune thyroiditis (AIT), GD and GO [3]. We and others have shown that TRAb persists at higher titers than non-smokers during treatment with methimazole [4]. In orbital tissues we have studied other risk factors for GO and found that smokers have increased expression of CYR61 [14]. Smoking is a risk factor for AIT and for other autoimmune diseases like rheumatoid arthritis, these patients show higher activity parameters in smokers [19].

It is well established that HLA and CTLA-4 are associated with GD, but there is lack of a specific genotype to GO. However, in a previous microarray we have shown that a matrix protein, CYR61, is increased in patients with severe GO in the active phase with a decrease in the chronic phase [12]. In another microarray of orbital tissue, we have also shown that CYR61 is preferentially increased in smokers compared with non-smokers [14]. In a genotyping study, we have demonstrated an increased risk of developing GO in smokersassociated with CYR61 [13].

In the present study, we have found a new association of TRAb titers elevated above the median and rs231775 in *CTLA-4*. The variant is located in exon 1 + 49 and it was shown that the A to G substitution at this location is linked with GD [20]. A study by Ban et al. found that the genotype GG in the CTLA4 A/G_49_ SNP resulted in a reduced increase in T-cell proliferation compared with the AA genotype [21].

Furthermore a previous study showed that a gene polymorphism in CTLA-4 contributes to the pathogenesis of GD because when peripheral blood cells were incubated with a monoclonal anti-human CTLA-4 monoclonal antibody resulted in increased blood cell proliferation [22]. It has also been demonstrated that a splice variant of CTLA-4 results in the release of a soluble form of CTLA-4 with increased levels in serum from patients with GD and that this soluble form bind to CD80/CD86, resulting in inhibition of T-cell activation [23]. Later, it was shown that increased levels of soluble CTLA-4 correlates with the severity of GO and that a genetic variation in the CTLA-4 gene region partially determines the level of its soluble form [24]. Other autoimmune conditions, like myasthenia gravis, SLE, coeliac disease and type 1 diabetes, are associated with soluble forms of CTLA-4, which has been reviewed by Saverino et al. [25]. However, no study describes an association between a SNP in CTLA-4 and increased autoantibody titers as in our study with increased titers of TRAb.

To conclude, we have demonstrated that the increase in TRAb titers after treatment with radioiodine of GD patients without GO is associated with later development of GO compared to a group that does not increase in TRAb titers. Also we have demonstrated that a genetic variation in CTLA-4 is associated with higher titers of TRAb after treatment with radioiodine.
